# Influence of Minor Alloying Element Additions on the Crack Susceptibility of a Nickel Based Superalloy Manufactured by LPBF

**DOI:** 10.3390/ma14195702

**Published:** 2021-09-30

**Authors:** Mireia Vilanova, Mari Carmen Taboada, Ana Martinez-Amesti, Andrea Niklas, Maria San Sebastian, Teresa Guraya

**Affiliations:** 1 LORTEK Technological Centre, Basque Research and Technology Alliance (BRTA), Arranomendia Kalea 4A, 20240 Ordizia, Spain; mctaboada@lortek.es (M.C.T.); msansebastian@lortek.es (M.S.S.); 2 Minning and Metallurgical Engineering and Materials Science Department, University of the Basque Coutry UPV/EHU, Rafael Moreno “Pitxitxi”, 2, 48013 Bilbao, Spain; teresa.guraya@ehu.eus; 3 SGIker, Advanced Research Facilities, UPV/EHU, Avda. Tolosa 72, 20018 Donostia-San Sebastian, Spain; ana.martinez@ehu.eus; 4 Fundación AZTERLAN, Basque Research and Technology Alliance (BRTA), Aliendalde Auzunea 6, 48200 Durango, Spain; aniklas@azterlan.es

**Keywords:** laser powder bed fusion (LPBF), nickel superalloy, solidification cracking, minor alloying elements influence

## Abstract

Inconel 738LC (IN738LC) is a nickel-based superalloy specially used in the hot section components of turbine engines. One of its main drawbacks relies on the cracking susceptibility when it is manufactured by laser powder bed fusion (LPBF). This paper analyzes the influence of minor alloying element concentration on cracking tendency of IN738LC superalloy manufactured by LPBF. For that objective, samples were manufactured using two powders, which presented different minor alloying elements concentration (Si, Zr and B). It was shown that the samples crack tendency was very different depending on the powder used for their manufacturing. In fact, the measured crack density value was 2.73 mm/mm^2^ for the samples manufactured with the powder with higher minor alloying elements concentration, while 0.25 mm/mm^2^ for the others. Additionally, a special emphasis has been put on elemental composition characterization in cracked grain boundaries in order to quantify possible Si or Zr enrichment. It has been also studied the differences of solidification ranges and grain structures between both samples as a consequence of different minor alloying elements concentration in order to analyze their effect on crack susceptibility. In this sense, Scheil-Gulliver simulation results have shown that samples with higher Si and Zr contents presented higher solidification range temperature. This fact, as well as an increase of the presence of high angle grain boundaries (HAGB), leaded to an increment in the crack formation during solidification. Therefore, in this research work, an understanding of the factors affecting crack phenomenon in the LPBF manufactured IN738LC was accomplished.

## 1. Introduction

Additive manufacturing (AM) is a manufacturing method in which parts are built by material addition layer-by-layer; in fact, each layer is a cross-section of the original computer aided design (CAD) data [1]. Among AM technologies, laser powder bed fusion (LPBF) is one of the most widely used. During LPBF process, a laser beam selectively melts the powder deposited over a building platform following the CAD model [2]. Additionally, several process parameters as laser power (P), laser scanning speed (v), hatch distance (h), layer thickness (t) or scan strategy (θ) have to be defined prior to samples manufacturing. In this aspect, combining some process parameters, a factor known as energy density (E_v_) can be calculated, which estimates the energy applied to the system during manufacturing process [3].

(1) $$E_{d}=\frac{P}{v\cdot h\cdot t}$$

One of the main drawbacks of LPBF technology is the limited materials that can be manufactured without defect formation. In this context, the most commonly manufactured materials by LPBF are AlSi_10_Mg [4] and Scalmalloy^®^ [5] aluminum alloys, IN718 [6], Hastelloy X [7] and Inconel 625 [8] nickel alloys, Ti_6_Al_4_V [9] titanium alloy and 316L stainless steel [10]. Nevertheless, great efforts have been conducting in order to expand the materials pallet that can be manufactured by LPBF. 

Inconel 738LC (IN738LC) is a nickel based superalloy with high creep properties and hot corrosion resistance up to 980 °C temperature [11]. Based on these extraordinary characteristics, it is commonly used in the hottest section of stationary and non-stationary gas turbines. The crystal structure of the superalloy consists of a face centered cubic (FCC) matrix strengthened by γ’ precipitates, which have a nominal composition of Ni_3_(Al,Ti) [12]. Furthermore, IN738LC superalloy contains a wide range of alloying elements such as Cr, W, Mo and Ta for solid solution strengthening, C for carbide formation and Zr, Si and B for grain boundary strengthening [13]. However, IN738LC superalloy presents high crack susceptibility and it is very challenging to study the causes that induce cracking in the LPBF manufactured samples [14]. In fact, different types of cracking, such as liquation cracking, solidification cracking or strain age cracking may occur in the IN738LC superalloy. Liquation cracking refers to the cracking originated by the reaction of a secondary phase with the matrix, which forms a non-equilibrium film in the particle/matrix interface. In the IN738LC superalloy, the secondary phases that could promote liquation cracking are MC carbides, borides, sulphocarbides, γ/γ’ eutectic or coarse γ’ precipitates [15]. This kind of cracks form by the remelting of the secondary phases located at grain boundaries; thus, these cracks appear at Heat Affected Zone (HAZ) grain boundaries. With respect to solidification cracking, it occurs in the solidification final stage when solute-rich liquid becomes trapped between two solid interfaces [16]. Due to tensile stresses generated by the solidification process, decohesion of liquid films may occur leading to the formation of cracks at grain boundaries. Finally, the cracking mechanism known as strain age cracking occur due to the excessive precipitation of γ’ phase during heat treatment of the superalloy. This precipitation implies a ductility reduction that together with residual stresses could lead to crack formation [17]. Nevertheless, in the LPBF manufactured as-built IN738LC superalloy, it is mostly established that the crack formation is mainly due to solidification cracking mechanism [18,19]. Additionally, some published research works point out the influence of powder particle size distribution on the crack formation in additively manufactured parts [20,21]. It is stated that when very fine powder particles are used, the powder experienced severe agglomeration and the processing of the part becomes difficult. In addition, smaller particle sizes melts with lower energy density, which implies that the remaining energy density increase the thermal gradients leading to the formation of higher residual stresses in the manufactured part.

According to the literature, there are several factors that could influence solidification crack formation in the IN738LC superalloy. Among these factors, it seems that the most important ones are the superalloy composition and the superalloy final grain structure. In this sense, M. Cloots et al. [22] investigated the composition of a grain boundary by atom probe tomography (APT) and observed that it was enriched in Zr and B alloying elements. This finding concluded that during LPBF processing, Zr and B elements had the effect of lowering the solidus temperature of the superalloy, which induced an increase in the solidification range. This temperature range is also known as critical temperature range (CTR), where the material is more susceptible to solidification crack formation. Thus, the authors identified the increase in Zr and B microsegregation at grain boundaries, which implies the generation of higher shrinkage strains, as the cause of solidification crack formation. Comparatively, Hariharan et al. [18] identified also by APT that Zr and Si were only enriched at high angle grain boundaries (HAGB) and they stated that these elements enrichment promoted an increase in the solidification range of the superalloy. However, they concluded that as the same Zr and Si concentration was observed in cracked and non-cracked HAGB, Zr and Si enrichment at grain boundaries was not the main reason for solidification cracks formation. Additionally, R. Engeli et al. [23] manufactured IN738LC samples by LPBF using several powder batches with different Si concentrations. Actually, samples manufactured with each powder batch presented different crack density values, obtaining lower crack densities in the samples with reduced Si content. Consequently, these authors stated that the maximum Si content in IN738LC superalloy should be below 0.03% in order to manufacture crack free samples. Nevertheless, they did not describe in detail the role of Si in the crack formation mechanism. Similarly, Zhu et al. [24] investigated the effect of Si addition and solidification rate in a directionally solidified IN738 superalloy. They demonstrated that increasing Si content and also solidification rate, leaded to a solidification range increase, which affects the morphology of solid–liquid interface. Furthermore, these authors observed that Si and Zr were segregated to the interdendritic region due to their low partition coefficients. 

As previously defined, the other factor that affects crack formation in LPBF manufactured samples is the grain structure, which is defined by the grains size, morphology and misorientation. In the LPBF samples, the formation of large columnar grains in the building direction is favored due to the layer-by-layer manufacturing process [25]. It is well known that cracks are more prone to form in high angle grain boundaries, which have higher misorientation values. Generally, high angle grain boundaries have higher grain boundary energy, which implies lower coalescence temperatures during solidification. This effect leads to liquid accumulation up to lower temperatures in the grain boundaries, which increases the crack susceptible region known as mushy zone and generates localized strain at the grain boundaries [26]. In the research work of Chauvet et al. [27], the authors observed that hot cracks were only formed at high angle grain boundaries, which seems to be related to the grain boundary energy variation. Finally, some authors [18,26] studied the effect of minor elements microsegregation and grains misorientation simultaneously in the formation of solidification cracks. They concluded that minor elements microsegregation was favored in the high angle grain boundaries, leading to an increase of the mushy zone in these localized areas and promoting the formation of solidification cracks.

In this research work, two IN738LC powders with different Si, Zr and B minor alloying elements content were manufactured by LPBF technology. Therefore, the effect of these minor elements on concentration and grain structure on crack susceptibility of IN738LC superalloy has been studied. A special emphasis has been put on elemental composition analysis of grain boundaries close to cracks. Furthermore, the crack susceptibility coefficient was calculated for samples with differences in minor alloying elements concentration and the importance of grain boundaries misorientation on crack formation was highlighted.

## 2. Materials and Methods

### 2.1. Materials

Two IN738LC powders produced by gas atomization were used as feedstock material for LPBF manufacturing. One batch was supplied by Aubert and Duval (A&D) from Eramet Group, whereas the other was supplied by LPW Technology Ltd. from Carpenter Technology Innovation (Philadelphia, United States). Table 1 presents the chemical composition of both powders measured by induced coupled plasma (ICP) technique and by LECO for carbon element. In the case of A&D powder, Si content is in range of the lower Si value of Engeli et al. [23], Zr content is 50% lower than in the LPW powder and B is an order of magnitude lower than in the LPW powder. 

**Figure 1:**
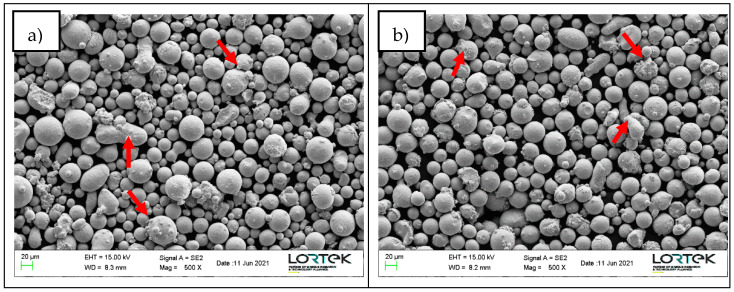
Powder particles SEM images indicating satellites and irregular particles with arrows; (**a**) A&D powder and (**b**) LPW powder.

Powder particles morphology was analyzed by scanning electron microscopy as shown in Figure 1. The particles of both powders present a generally spherical morphology; however, some of the particles have satellites attached to their surface. Additionally, in the A&D powder more irregular particles can be found. Apart from particles morphology, powder particle size distribution (PSD) was measured by image analysis. The obtained D10, D50 and D90 results are summarized in Table 2.

### 2.2. LPBF Process Parameters

Samples with the dimensions described in the image of Table 3 were manufactured in order to ease the extraction from the platform. The samples were manufactured with a Renishaw RenAM 500Q machine, which employs 4 Yb-fiber lasers in pulsed or continuous mode with a beam diameter of 85 µm and a maximum laser power of 500 W. Based on the results obtained in a preliminary research work [28], samples were processed using laser continuous mode and the process parameters described in Table 3. Actually, 90° scan strategy, which is defined as the rotation of layers, was selected because it was observed in the previous work that crack formation was favored in this scan strategy. The obtained energy density value is inside the optimal range defined by Vilanova et al. [28]. 

### 2.3. Samples Characterization

Samples were cut in the building direction (x–z plane) for defect quantification and microstructural analysis. Subsequently, they were grinded up to 2500 µm SiC paper and polished with 6, 3 and 1 µm diamond paste. Grain structure was revealed by chemical etching for few seconds with this specific reagent: 10 mL CH_3_COOH + 10 mL HNO_3_ + 15 mL HCl.

Porosity and cracking quantification were performed in non-etched samples to evaluate possible differences due to minor alloying elements addition. Porosity was analyzed by image analysis using the optical microscope (GX51 Olympus) and the AnalySIS Docu software. In fact, an area of 10 mm^2^ was studied in each sample and then the area porosity percentage was calculated. In order to dismiss the presence of cracks in this step, a circularity threshold value of 0.7 was established. Cracks quantification was carried out following the methodology described by Carter et al. [29] and the cracks were measured using the optical microscope and ImageJ software.

Additionally, samples were characterized using the Zeiss Ultra Plus Scanning Electron Microscope (FEG-SEM) in secondary electron (SE) mode at 15 kV to analyze the morphology and particle size. Furthermore, inverse pole figure (IPF) maps were measured by electron backscattered diffraction (EBSD). Both analyses were performed with Aztec, Oxford Instruments, NanoAnalysis software and for EBSD map, the scanned area was 300 × 300 µm^2^ using 1 µm step size and noise reduction was performed up to six neighbors. 

**Figure 2:**
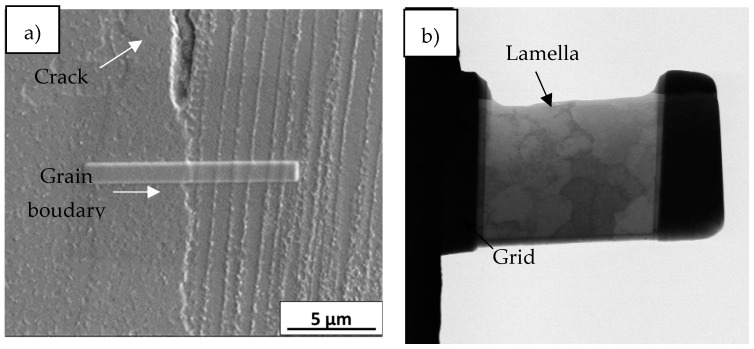
(**a**) Lamella extraction at a grain boundary and (**b**) lamella.

Transmission electron microscopy (TEM) was used to investigate more accurately the elemental composition at grain boundary. A lamella of ~50 nm thickness was prepared in front of a crack tip, as illustrated in Figure 2, via standard lift-out protocol using a Dual beam Helios 650 model, which consists of a 30 kV field-emission scanning electron column with 0.9 nm resolution and a 30 kV Ga focused ion beam (FIB). The lamella was analyzed using a FEI Titan3 Themis Transmission Electron Microscope (TEM) at 200 kV in the scanning transmission electron microscopy mode (STEM). This double aberration-corrected TEM incorporates advanced optical elements to compensate the intrinsic aberrations of both the objective and condenser lenses. Additionally, the TEM incorporates X-Ray energy dispersive spectroscopy (EDS) analysis (Seper-X G2) using four window-less SDD detectors providing a solid angle ≈ 0.7 srad. The maps were 512 × 512 and were obtained using 300 pA current intensity. Velox software was used for the composition maps acquisition and processing. 

## 3. Results

### 3.1. Analysis of Defects

**Figure 3:**
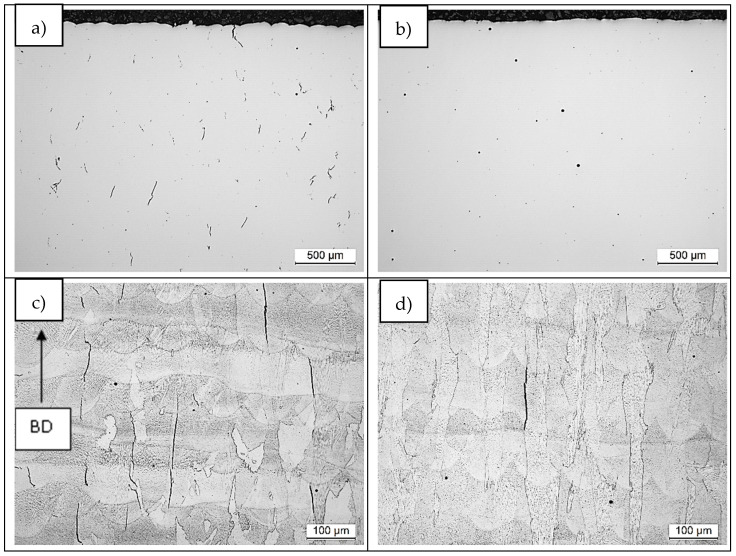
X–Z plane of both samples: (**a**,**c**) LPW and (**b**,**d**) A&D.

Samples were manufactured using both IN738LC compositions and the process parameters summarized in Figure 3. Analysis of defects by means of pores and cracks were characterized in both samples. Area porosity percentage value was 0.08% and 0.12% for LPW and A&D samples, respectively (Figure 3a,b). Actually, it is important to highlight that pores had random distribution and presented spherical morphology; therefore, they were probably formed due to gas entrapment during manufacturing process. 

With respect to crack tendency, crack density was measured also in both samples and the obtained values were 2.73 and 0.25 mm/mm^2^ for LPW and A&D samples. As shown in Figure 3a,b, much more cracks were formed in the LPW sample; which has a higher content of Si, Zr and B minor alloying elements. Apart from compositional differences, LPW sample also presents a lower particle size than A&D sample. As stated in the introduction part, this fact can also increase the crack tendency of the alloy due to residual stresses formation. A more detailed analysis was performed in etched samples to reveal the grain structure (Figure 3c,d), which shows three characteristics of the cracks: they go through several layers, they are parallel to building direction (BD) and they form at grain boundaries. The location of cracks will be analyzed in more detail later. Looking carefully the last layer of both samples, which have a depth of 150 µm approximately, the presence of some cracks is observed in the LPW sample, but not in the case of A&D sample. The presence of cracks in the last layer is indicative of solidification cracking because there is no reheating in the last layer to promote liquation cracking.

### 3.2. Grain Structure

**Figure 4:**
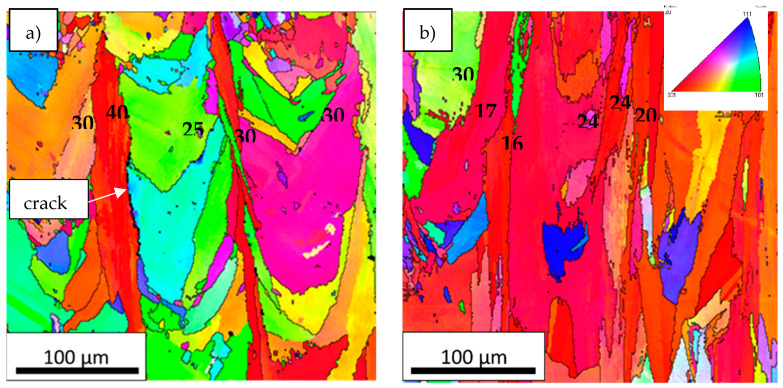
IPF images of (**a**) LPW and (**b**) A&D samples in building direction.

As it has been explained in the introduction, the typical grain structure of LPBF samples consists of large columnar grains [30]. Actually, Figure 4a,b illustrate the inverse pole figure (IPF) maps of LPW and A&D samples in which grains are defined with >15° grain boundary misorientation. Although the grains of both samples have columnar structure, there are visible differences between the LPW and A&D samples grain structure. In fact, LPW grains present a mean area of 500 ± 100 µm^2^ and an aspect ratio of 3 ± 1, whereas A&D grains mean area is 687 ± 70 and aspect ratio 4 ± 1. Thus, compared to LPW grains, A&D grains are more elongated and present a higher area. Comparing both IPF maps, it is obvious that the A&D sample exhibits more texture in <001> direction than LPW sample. Additionally, in the LPW IPF map, a crack is observed at a grain boundary with 40° misorientation between adjacent grains, which evidences that crack formation occurs intergranularly [16]. This effect is also visible in Figure 3c,d, where cracks formation also occurred at grain boundaries. In Figure 4a,b several grain boundary misorientation values are presented, but it must be highlighted that only one crack is observed in the IPF map of LPW sample (Figure 4a). In fact, the formation of this crack occurs in the grain boundary with 40° misorientation, which is the higher measured misorientation value in that sample. On the other hand, cracks were not observed in the IPF map of A&D sample (Figure 4b). 

### 3.3. Grain Boundary Elemental Composition Analysis

**Figure 5:**
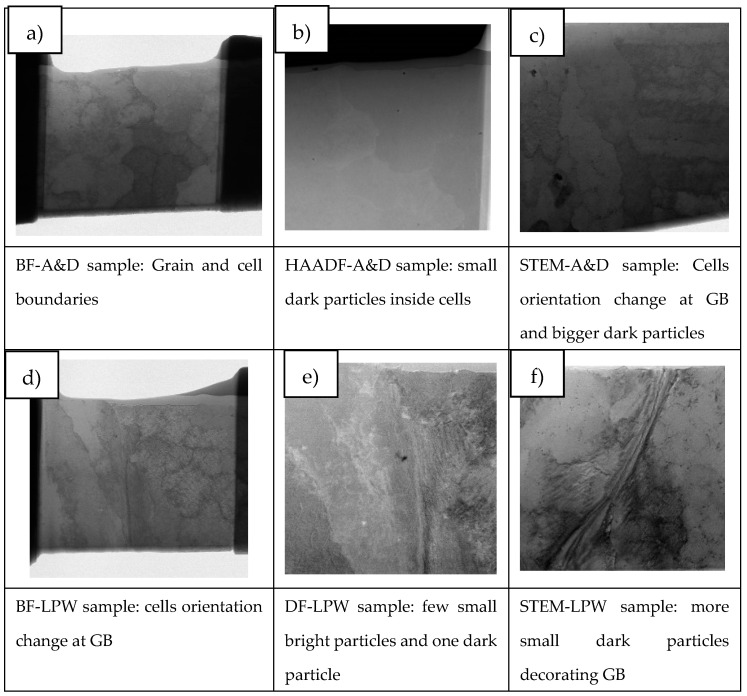
Different mode images of the lamellas lifted out from A&D and LPW samples: (**a**) BF of A&D sample, (**b**) A&D sample image obtained by HAADF detector, (**c**) A&D sample image in the scanning (STEM) mode, (**d**) BF of LPW sample, (**e**) LPW sample obtained by HAADF detector and (**f**) LPW sample image in the scanning (STEM) mode.

As explained in the experimental section, several lamellas were obtained by FIB technique in order to analyze the LPW and A&D samples grain boundary elemental composition by TEM. Some representative images of the grain structure of both samples are shown in Figure 5. TEM images were acquired using different detectors to highlight specific features of the microstructure as described under each image. Figure 5a,d are bright field (BF) TEM images of A&D and LPW samples, Figure 5b,e are TEM images obtained with the high-angle annular dark-field imaging (HAADF) detector of A&D and LPW samples and finally, Figure 5c,f are images of A&D and LPW samples obtained with the scanning (STEM) mode.

**Figure 6:**
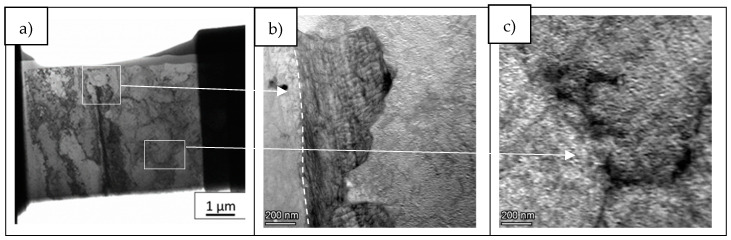
**(a)** LPW lamella, (**b**) grain boundary and (**c**) cell boundary.

**Figure 7:**
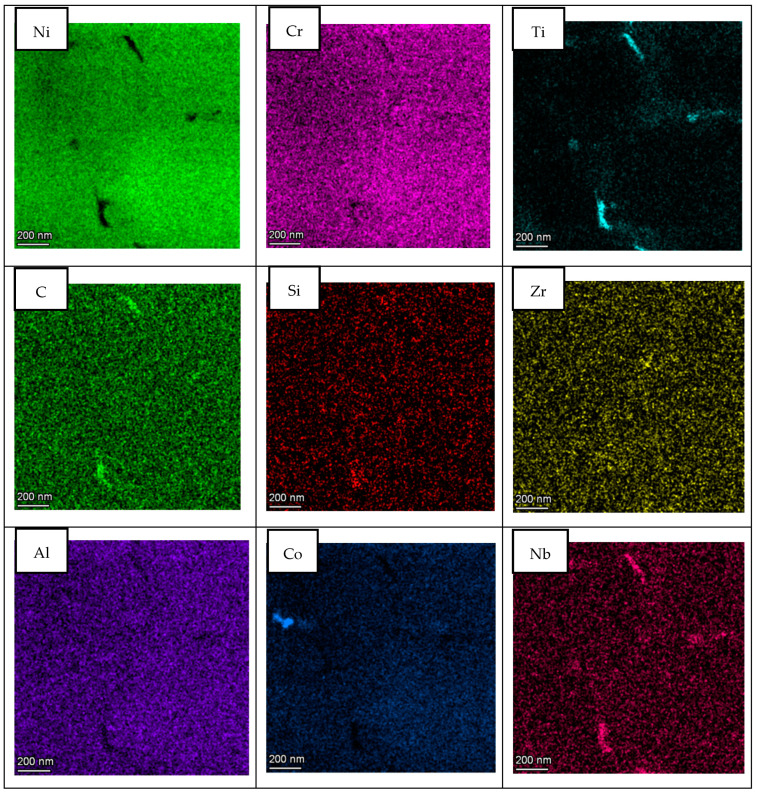
STEM EDS compositional maps of the LPW sample grain boundary.

In order to study segregation phenomena at cell and grain boundaries, the elemental composition maps were obtained by BF-STEM EDS in all cases. Figure 6 presents the area of the LPW sample analyzed and Figure 7 shows the elemental composition maps at the grain boundary in which there is an enrichment of Ti, C, Si and Nb elements in some specific zones and a depletion of Ni and Al. This enrichment or depletion of elements at the grain boundary is due to microsegregation process; in fact, other authors have also obtained similar results [18,24]. The difference in element distribution is based on each element partition coefficient; actually, Ti, Si and Nb have partition coefficients lower than one, which indicates that they were enriched in the liquid [23]. However, Al has a partition coefficient higher than one, indicating that it was mostly enriched in the solid. The dark particle visible at the top left in the image has resulted to be rich in Fe and mostly in Co. Due to its irregular-globular shape it can be associate to a Laves particle. 

**Figure 8:**
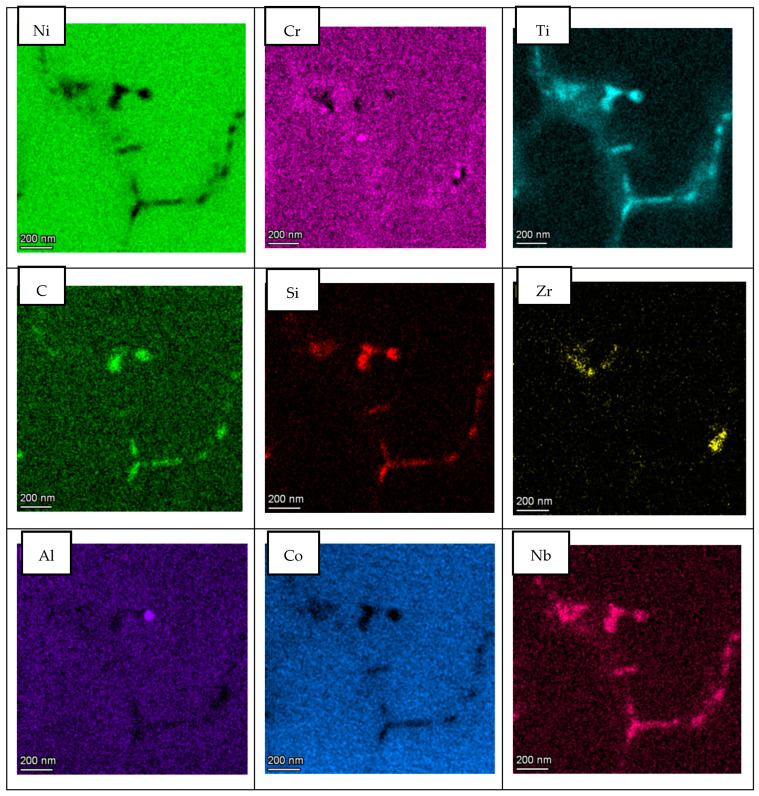
STEM EDS compositional maps of the LPW sample cell boundary.

Apart from these types of segregations, other authors have also observed the presence of titanium carbides in GB of IN738LC samples [18]. Our samples do not show presence of particles in all locations where segregation is noticeable. To illustrate that segregation is a consequence of the different partition coefficients and not to carbide formation during solidification, an additional elemental composition map was performed in the LPW sample. Figure 8 shows a detail of the cell boundary and the corresponding maps. Segregation of the same elements is clearly visible without any carbide or other particle associated. In fact, the formation of second phases is not favored in LPBF process due to the high cooling rates, which implies that the majority of alloying elements will be in solid solution state. 

**Figure 9:**
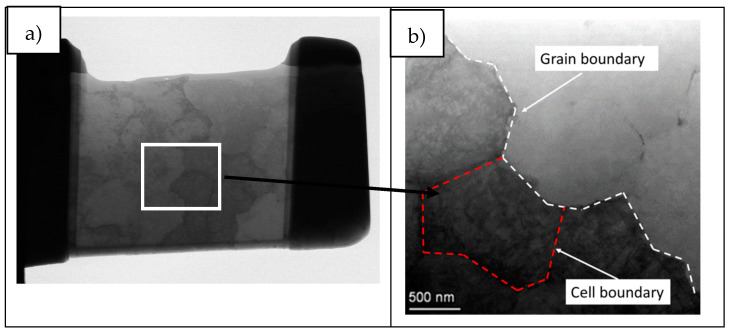
(**a**) A&D lamella and (**b**) magnification of grain and cell boundary.

**Figure 10:**
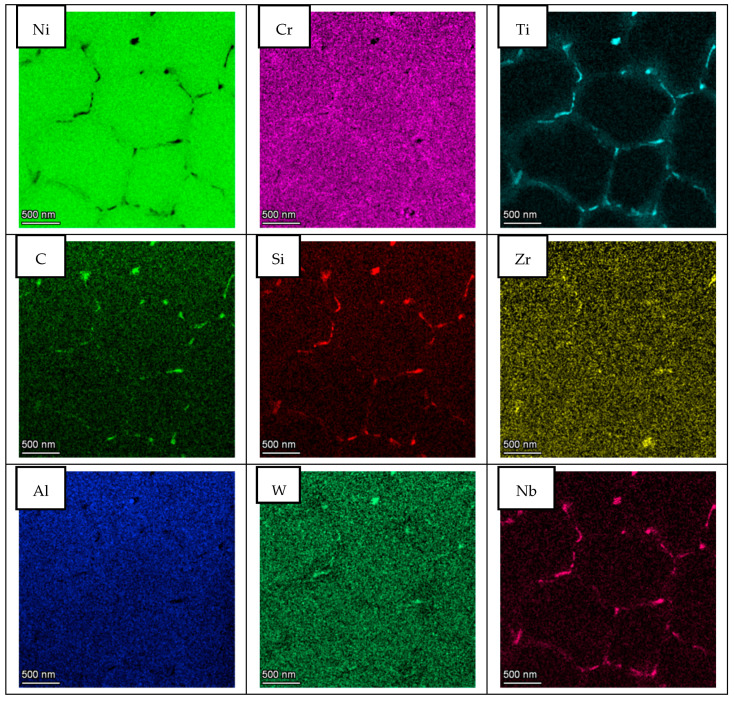
STEM EDS compositional maps of the A&D sample grain and cell boundaries.

With respect to A&D sample, the same analysis as for LPW sample was performed; however, in this case, grain and cell boundaries were localized in the same picture as shown in Figure 9. The results presented in Figure 10 are very similar to the ones obtained for the LPW sample; in fact, there is an enrichment of Ti, Si, Zr, C and Nb elements at grain and cell boundaries whereas there is a depletion of Ni and Cr. 

**Figure 11:**
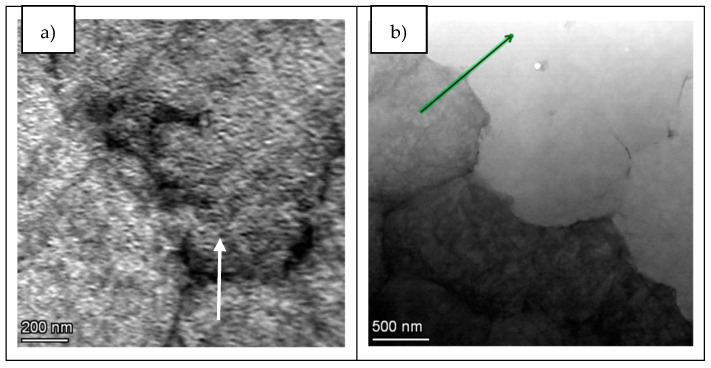
Identification of the zones for line-scans acquisitions: (**a**) LPW and (**b**) A&D.

With the aim of performing a more localized composition analysis, TEM EDS line-scans were performed in LPW sample cell boundary and in A&D sample grain boundary. In fact, the selected zones are presented in Figure 11, where the arrows indicate the location of the line-scan measurements.

**Figure 12:**
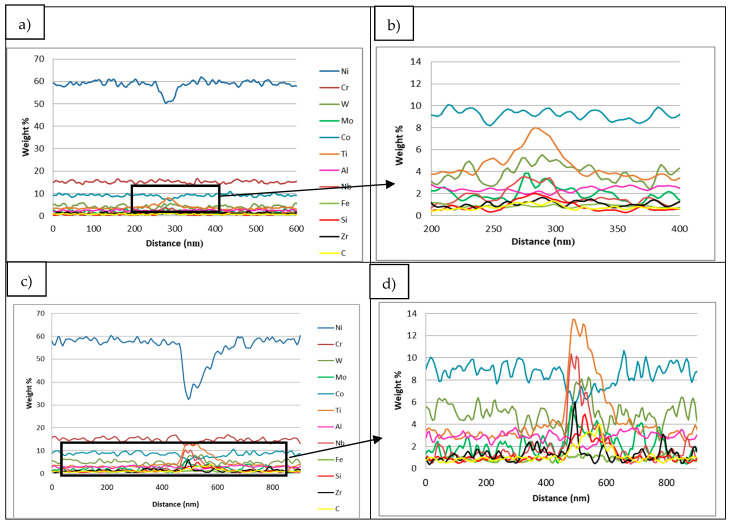
(**a**) LPW sample cell boundary line-scan, (**b**) zoom of LPW sample cell boundary line-scan and (**c**) A&D sample grain boundary line-scan and (**d**) zoom of A&D sample grain boundary line-scan.

In this aspect, as shown in Figure 12, both samples present similar composition at cell and grain boundaries, confirming the results obtained in the EDS maps. Actually, at cell and grain boundaries, there is a clear depletion of Ni while these zones are principally enriched in Ti, Nb and W elements. Furthermore, a slight increase of Si and C elements is also visible at the cell and grain boundaries’ locations. Thus, the enrichment of these elements at cell and grain boundaries evidenced that there is a segregation of certain elements at these locations. However, the segregation of Si and Zr at these zones is not as important as previously expected. 

## 4. Discussion

### 4.1. Effect of Minor Alloying Elements on Crack Susceptibility

Two different IN738LC compositions were employed to manufacture samples by LPBF technology. After chemical composition analysis, it was observed that the IN738LC powder supplied by LPW presented higher concentration of Si, Zr and B elements, which are supposed to increase the cracking tendency of this superalloy. Actually, it was experimentally demonstrated that LPW sample presented much more cracks than A&D sample, which indicates that minor additions of Zr, Si and B play an important role on crack tendency of IN738LC superalloy. In the literature, some authors have analyzed the effect of composition on cracking tendency by atom probe tomography (APT) [18,19,27]. They stated that at the last solidification stage, grain boundaries were enriched in some alloying elements, which lead to a higher solidification range and cracking susceptibility. Thus, in this research work, STEM X-EDS was used to qualitatively analyze the microsegregation of alloying elements at grain and cell boundaries. It was observed that depending on the partition coefficient, some elements such as Ti, Nb, Si, C and Zr with partition coefficients lower than one were segregated to grain and cell boundaries. On the other hand, elements with partition coefficients higher than one were depleted in these zones. Nevertheless, the same alloying elements segregation was observed at grain and cell boundaries for the LPW and A&D powders respectively. Thus, it was not possible to establish any difference between both powders in this aspect. 

**Figure 13:**
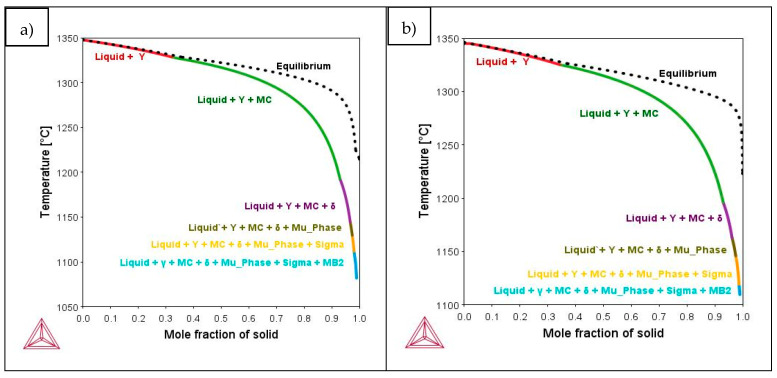
Scheil-Gulliver simulations of (**a**) LPW and (**b**) A&D samples.

Additionally, S. Kou [31] suggested a criterion that links the solidification cracking susceptibility of an alloy with the slope of the T (temperature) vs. fs^1/2^ (fs refers to solid fraction) curve in the range between 0.94–0.97 solid fraction (f_s_). In order to verify if the studied cases satisfy this criterion, Scheil-Gulliver simulations were performed with ThermoCalc software and TCNI10 database (Figure 13). Scheil-Gulliver simulation, which only considers diffusion in liquid state, was selected due to non-equilibrium solidification during LPBF [32]. Applying the criterion proposed by S. Kuo [31], it was verified that LPW sample presents a slope of the T vs. f^1/2^ curve in the f_s_ range of 0.94–0.97 27% greater than A&D sample. Therefore, the samples investigated in this work follow the criterion previously described. 

Finally, in order to compare the crack susceptibility of both samples, crack susceptibility coefficient (CSC) was calculated based on Clyne and Davies research [33]. These researchers explained that a materials susceptibility to cracking depends on critical time periods during the solidification process. In addition, they stated that liquid feeding to interdendritic areas occurs at liquid volume fractions between 0.6 and 0.1, and defined the time between this range as time relaxation (t_R_). On the other hand, when the liquid volume is between 0.1–0.01, they established that material is in the vulnerable region and the time spent in this range is defined as t_V_. Thus, the relation between t_V_ and t_R_ results in the CSC, implying that a higher CSC results in higher crack tendency.

**Figure 14:**
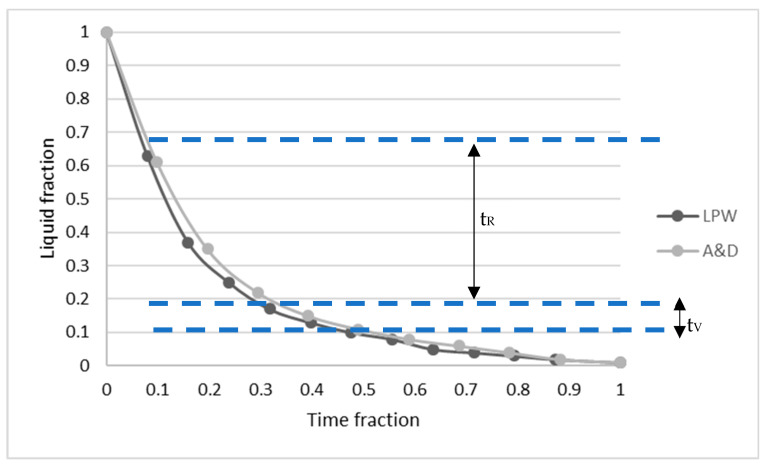
Curves obtained following Clyne and Davies [33] criterion for LPW and A&D samples.

In order to calculate CSC value, constant cooling rate heat flow mode was selected based on the rapid solidification occurred in LPBF technology. Firstly, LPW and A&D samples cooling rates (Ṫ) were calculated following the method described by Guraya et al. [34], where λ_1_ (primary dendrite arm space) must be measured. Secondly, the solidification time with respect to liquid fraction (f_L_) can be calculated (Figure 14) using the cooling rate and the solidification range of each sample, which was obtained with Scheil-Gulliver simulation. Finally, with the liquid fraction and time fraction during solidification, it is possible to calculate the t_V_ and t_R_ values; thus, the CSC value of each sample. Table 4 summarizes λ_1_, Ṫ, ΔT, t_V_, t_R_ and CSC values. Actually, LPW sample presents higher CSC value than A&D sample, which means that this superalloy will have higher cracking susceptibility as validated with experimental results.

### 4.2. Effect of Grain Structure

**Figure 15:**
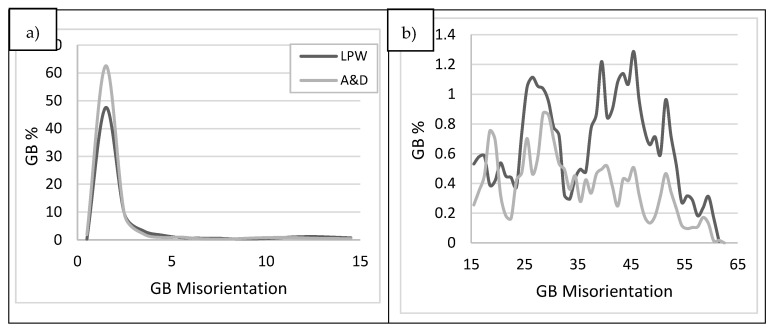
LPW and A&D samples grain boundary misorientation: (**a**) LAGB and (**b**) HAGB.

Apart from minor alloying elements segregation, in the literature it is suggested that grain boundary (GB) misorientation plays an important role in the cracking susceptibility of a material [35]. Based on IPF maps information, LPW and A&D samples GBs misorientations were measured as shown in Figure 15a,b. Actually, it was quantified that A&D sample contained more low angle grain boundaries (LAGB < 15° misorientation) than LPW sample (Figure 15a). Nevertheless, in the case of high angle grain boundaries (HAGB > 15°), as exhibited in Figure 15b, LPW sample contained more misoriented grain boundaries than A&D sample. This fact coincides with the higher cracking tendency of LPW sample, since more misoriented grains are more prone to cracking [27,36].

Rappaz et al. [37] discussed the higher crack tendency of HAGB compared to LAGB and related this fact with the dendrites coalescence behaviour. Actually, these researchers established the concept of attractive and repulsive boundaries based on the crystallographic misorientation. They stated that in the attractive GBs, secondary arms coalescence in the last stage of solidification; however, in the case of repulsive GBs, a liquid film remains stable at lower temperatures preventing the coalescence of secondary arms. In order to clarify the attractive and repulsive concepts, the researchers compared the grain boundary energy γ_gb_ to twice the solid-liquid interface energy γ_sl_. Therefore, when γ_gb_ < 2 γ_sl_, the liquid film will be unstable and coalescence between dendrites will occur, which is the case of attractive GB. On the other hand, in the repulsive GBs, γ_gb_ > 2 γ_sl_, the liquid film remains stable at lower temperatures; thus, grain boundary remains wetted until lower temperatures. This effect could explain the increase of crack formation in the LPW sample compared to A&D sample, based on the fact that LPW sample has GBs with higher misorientation than A&D.

**Figure 16:**
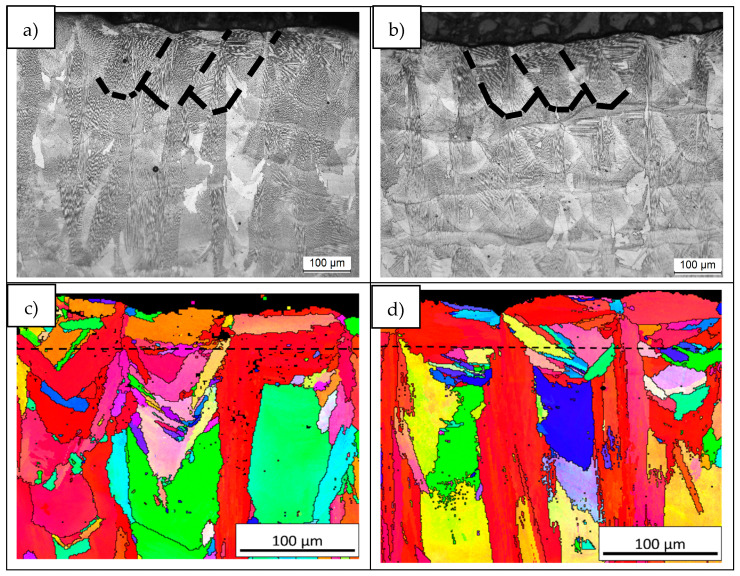
(**a**) LPW melt pools, (**b**) A&D melt pools, (**c**) IPF figure of LPW sample last layer and (**d**) IPF figure of A&D sample last layer.

Furthermore, melt pools from the last layer of LPW (Figure 16a) and A&D (Figure 16b) samples were investigated due to the fact that they are the unique zones that do not suffer any reheating process. Thus, ~50 µm of the last layers were analyzed (dashed lines in Figure 16c,d) because they are not affected by previous melted material. After the evaluation of both samples last layer IPF images, it was verified that LPW sample last layer exhibited more grains, which were smaller and presented higher misorientation between adjacent grains compared to the ones observed in the A&D sample last layer. Indeed, grain boundaries misorientation values in the last layer of LPW sample were in the range of 30°–50°, whereas for the A&D sample were between 15°–25°. This fact indicates that the grain structure of LPW and A&D samples is different from the first stages of solidification, leading to a microstructure with more grains and less texture in the case of LPW samples. This finding is related with the formation of stray grains in single crystals; in fact, de Busaac et al. [38] described that misorientation always results in an increasing risk of stray crystal formation. The formation of stray grains is also connected with the presence of repulsive or divergent grain boundaries explained in the previous paragraph. 

Thus, a higher content of minor alloying elements implies an expansion of the alloy solidification range, which favors the formation of repulsive grain boundaries. Indeed, these repulsive grain boundaries lead to the formation of adjacent grains with higher misorientation and also facilitate the stray grains formation. As a result, a less textured grain structure with smaller and more misoriented grains is formed. Thus, these factors acting together involve higher crack susceptibility.

## 5. Conclusions

In this research work, it was presented that using the same LPBF process parameters, LPW sample, which contains higher Si and Zr content, exhibited much more solidification cracks than A&D sample. This fact implies that minor alloying elements addition has huge influence on crack formation. 

In all the cases, solidification cracks were developed at grain boundaries; therefore, composition map measurements were performed in these zones using TEM EDS. The results showed that there were no significant segregation differences between LPW and A&D samples at grain and cell boundaries. Nevertheless, Scheil-Gulliver simulations indicated that LPW sample presented higher solidification range than A&D sample, which is known to be an important factor affecting crack formation. Furthermore, crack susceptibility coefficient was calculated using the results from the simulation. In this sense, the CSC value obtained for LPW sample was higher than the one obtained for A&D sample. Thus, LPW sample will be more prone to cracking than A&D sample because it spends more time in the vulnerable region. 

Additionally, the effect of samples grain structure was investigated with respect to cracking tendency. It was observed that LPW sample presents GBs with higher misorientation than A&D sample. This is an indicative that samples with more misoriented grains are more prone to cracking probably due to the existence of liquid films at lower temperatures. This fact provokes that GBs remain wetted during more time, which increases the possibility of crack formation. 

Therefore, it was concluded that controlling minor alloying elements composition of IN738LC is critical to avoid crack formation during LPBF manufacturing. Furthermore, it was observed that minor alloying elements concentration affects both, sample grain structure and solidification range, which are factors that increase crack formation tendency. 

## Figures and Tables

**Table 1:** Chemical composition (weight %) of Aubert and Duval and LPW IN738LC powders.

	Ni	Al	Ti	Cr	Co	Mo	Nb	Ta	W	Fe	Si	Zr	C	B
**A&D**	Bal.	3.6	3.3	15.8	8.6	1.8	0.8	1.8	2.7	0.04	0.02	0.04	0.10	0.0009
**LPW**	Bal.	3.3	3.3	15.7	8.1	1.7	0.9	1.7	2.7	0.02	0.03	0.06	0.11	0.011

**Table 2:** PSD for A&D and LPW powders.

	D10 (µm)	D50 (µm)	D90 (µm)
**A&D**	33	49	64
**LPW**	22	33	44

**Table 3:** Sample dimensions and LPBF process parameters.

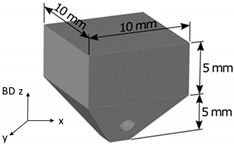	Process parameter	
	Laser Power (W)	230
	Scan speed (mm/s)	900
	Hatch distance (mm)	0.1
	Layer thickness (µm)	60
	Scan strategy (^o^)	90
	Energy density (J/mm^3^)	43

**Table 4:** Calculated values of LPW and A&D samples for CSC calculation.

Sample	λ_1_ (µm)	Ṫ (K/s)	ΔT (K)	t_V_ (s)	t_R_ (s)	CSC
LPW	0.55	4236696	266	0.52	0.38	1.36
A&D	0.54	4565959	235	0.50	0.39	1.27

## Data Availability

The data presented in this study are available on request from the corresponding author.
